# Plant-Pathogen Interaction, Circadian Rhythm, and Hormone-Related Gene Expression Provide Indicators of Phytoplasma Infection in *Paulownia fortunei*

**DOI:** 10.3390/ijms151223141

**Published:** 2014-12-12

**Authors:** Guoqiang Fan, Yanpeng Dong, Minjie Deng, Zhenli Zhao, Suyan Niu, Enkai Xu

**Affiliations:** Institute of Paulownia, Henan Agricultural University, 95 Wenhua Road, Jinshui District, Zhengzhou 450002, China; E-Mails: dongdyp@163.com (Y.D.); dengmj1980@126.com (M.D.); zhaozhl2006@126.com (Z.Z.); suyanniu_happy@126.com (S.N.); xuenkai19831206@163.com (E.X.)

**Keywords:** *Paulownia fortunei*, *Paulownia* witches’ broom (PaWB), phtoplasma, *de novo* assembled transcriptome, disease resistance gene

## Abstract

Phytoplasmas are mycoplasma-like pathogens of witches’ broom disease, and are responsible for serious yield losses of *Paulownia* trees worldwide. The molecular mechanisms of disease development in *Paulownia* are of considerable interest, but still poorly understood. Here, we have applied transcriptome sequencing technology and a *de novo* assembly approach to analyze gene expression profiles in *Paulownia fortunei* infected by phytoplasmas. Our previous researches suggested that methyl methane sulfonated (MMS) could reverse the effects of the infection. In this study, leaf samples from healthy, infected, and both infected and methyl methane sulfonate treated plants were analyzed. The results showed that the gene expression profile of *P. fortunei* underwent dramatic changes after *Paulownia* witches’ broom (PaWB) phytoplasma infection. Genes that encoded key enzymes in plant-pathogen interaction processes were significantly up-regulated in the PaWB-infected *Paulownia*. Genes involved in circadian rhythm and hormone-related genes were also altered in *Paulownia* after PaWB infection. However, after the PaWB-infected plants were treated with MMS, the expression profiles of these genes returned to the levels in the healthy controls. The data will help identify potential PaWB disease-resistance genes that could be targeted to inhibit the growth and reproduction of the pathogen and to increase plant resistance.

## 1. Introduction

*Paulownia* witches’ broom (PaWB) disease is a devastating disease of *Paulownia* trees, which can result in significant economic losses over a large part of the world [1,2]. Infected plants develop a series of similar symptoms, such as witches’ brooms, short internodes, yellowing or reddening of leaves, phyllody, stunting and decline, virescence, sterile flowers and necrosis, and altered volatile production [3]. It has been reported that the disease is caused by an obligate biotrophic plant pathogen called phytoplasma, which belongs to the taxon “*Candidatus* Phytoplasma australiense” International Research Programme on Comparative Mycoplasmology (IRPCM), Phytoplasma Taxonomy Group 2004 [4]. Phytoplasma are mycoplasma-like organisms that reside in the phloem sieve cells of a plant and are transmitted by phloem-sucking insects [5]. They belong to the Bacteria Kingdom. After residence, they can travel systemically through the pores of the sieve plates and appear to accumulate mainly in roots, developing leaves, and flowers. Phytoplasmas have no cell wall and are strictly biotrophic; thus, they can survive only on living host cells and are quite difficult to study.

Studies of PaWB disease during the last 30 years have been focused mainly on the ecological and biological characteristics of the disease, while the mechanisms behind it are largely unknown. Although researchers recognize that understanding the mechanisms is important, studying them is difficult largely because phytoplasma have never been cultured [6]. Recently, our studies showed that the infected *Paulownia* seedlings could recover a healthy morphology by treatment with suitable concentration of methyl methane sulfonate (MMS), in which the phytoplasma could be removed [7,8,9].

Some molecular biology-based methods have been developed to detect the existence of phytoplasma [10,11] and more information about PaWB disease has been explored. However, a comprehensive view of the process has yet to be formed. Recently, next-generation sequencing has been used to study this complicated biological system. Mou *et al.* [12] conducted a transcriptome study on healthy and phytoplasma-infected *Paulownia tomentosa* × *Paulownia fortunei* samples using RNA-Seq. At about the same time, our team [13] reported a similar study. There are two main differences between these two studies: (1) the samples used in our research came from tissue cultured plants, while the samples used by Mou *et al.* were both tissue cultured plants and field grown ones. However, in our study, samples that had been treated with MMS were included; (2) the results of the two studies differ from each other. Different genes were found differentially expressed in the infected *Paulownia*. We proposed a hypothesis to interpret the possible functional roles of the identified differentially expressed genesin *Paulownia*-phytoplasma compatible interactions, while Mou *et al.* found that genes encoding key enzymes in cytokinin biosynthesis were significantly induced in the phytoplasma-infected samples, and genes involved in cell wall biosynthesis and degradation were largely up-regulated while genes related to photosynthesis were down-regulated in the phytoplasma-infected samples.

In the present research, we studied the effects of MMS on the morphological changes of *P. fortunei* seedlings with PaWB disease and found that the seedlings treated with 60 mg·L^−1^ MMS for more than 3 h showed no disease symptoms. After that, we performed a transcriptomic analysis using the Illumina/SolexaGAIIx platform and *de novo* assembly to investigate the differences among three *P. fortunei* samples: PF (healthy), PFI (phytoplasma-infected) and PFI-60 (phytoplasma-infected and 60 mg·L^−1^ MMS-treated). Our study demonstrated that dramatic changes in gene expression occurred in the phytoplasma infection process, while after MMS treatment, the expression profile of a large number of genes changed back to the profile of the healthy sample. Our study has provided genome-wide potential phytoplasma-resistant genes and inherent pathways that are candidates for further study. The results presented provide a comprehensive platform on which our understanding of the complicated biological process of plant-phytoplasma interactions can be built.

## 2. Results

### 2.1. Detection of Paulownia Witches’ Broom (PaWB) Phytoplasma in Paulownia Showing Disease Symptoms

**Figure 1:**
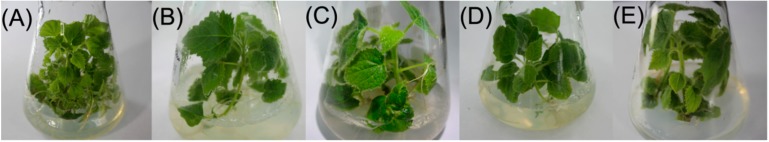
Methyl methane sulfonate (MMS) treated plantlets morphology. (**A**) *Paulownia* witches’ broom (PaWB)-infected plantlets, symptoms such as witches’ brooms were shown; (**B**) 20 mg·L^−1^ MMS; (**C**) 60 mg·L^−1^ MMS; (**D**) 100 mg·L^−1^ MMS; (**E**) Healthy plantlets.

**Figure 2:**
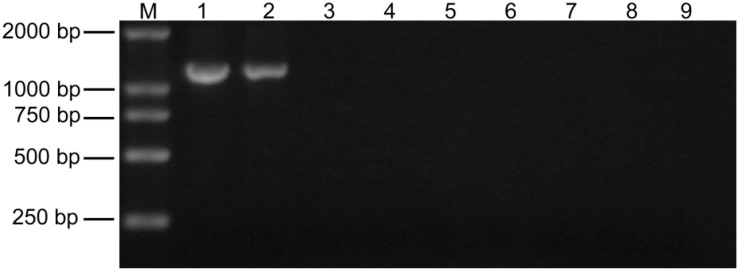
Phytoplasma 16SrDNA amplication in MMS treated plantlets. (**1**) PaWB-infected plantlets; (**2**) 20 mg·L^−1^ MMS; (**3**) 60 mg·L^−1^ MMS; (**4**) 100 mg·L^−1^ MMS; (**5**) Healthy plantlets; (**6**) ddH_2_O; (**M**) DNA Marker.

After PaWBphytoplasma infection, the *Paulownia* plants showed mainly typical disease symptoms, including witches’ broom, yellowing of leaves, smaller leaves, short internodes, phyllody, and sterile flowers. When the concentration of the MMS was less than 20 mg·L^−1^, the PaWB seedlings retained an unhealthy morphology. After 20–60 mg·L^−1^ MMS treatment, the PaWB seedlings regained a healthy morphology. When the concentration of MMS was above 60 mg·L^−1^, the growth of the PaWB seedlings was inhibited (Figure 1). The 16SrDNA of PaWB phytoplasma is 1.24 kb long [14]. During our study, fragments of the 16SrDNA were detected in the midribs of the PFI and PFI-20 (phytoplasma-infected and 20 mg·L^−1^ MMS-treated) samples, but not in the PF and PFI-60 samples (Figure 2). These results indicated that the PaWB infected seedlings recovered a healthy morphology after treatment with an optional concentration of MMS.

### 2.2. Transcriptome Sequencing and Assembly

The Illumina Solexa sequencing generated 57,846,728 high-quality paired-end reads, 58,451,828 high-quality paired-end reads, and 51,417,240 high-quality paired-end reads for the PF, PFI, and PTFI-60 libraries, respectively. The lengths of the reads were between 90 and 100 bp. All the high-quality reads in the three libraries were assembled into contigs, which were further assembled into unigenes. From the PF, PFI, and PFI-60 libraries, the assembly produced 116,749, 91,997, and 103,300 unigenes with average lengths of 1016, 864 and 1029 bp, and median lengths (N50) of 1963, 1715, and 1899 bp, respectively (Table 1). Together we obtained a total of 115,116 non-redundant unigenes (all unigenes) with a mean length of 1282 bp, and N50 of 2067 bp. Among these unigenes, 75,498 (65.58%) were longer than 500 bp, and 55,029 (47.80%) were longer than 1000 bp (Figure S1).

**Table 1:** Overview of the sequencing and assembly of the transcriptome of *P. fortunei*.

Statistics of Data Production	PF	PFI	PFI-60
Number of high-quality reads	57,846,728	58,451,828	51,417,240
GC percentage (%)	46.30%	46.39%	46.39%
Contigs	PF	PFI	PFI-60
Number of contigs	174,105	155,253	175,247
Average length of contigs (nt)	358	361	339
Length of N50 (bp)	670	740	654
Unigenes	PF	PFI	PFI-60
Number of unigenes	116,749	91,997	103,300
Length of N50 (bp)	1963	1715	1899
Average length of unigenes (bp)	1016	864	1029
All unigenes			
Number of all unigenes	115,116		
Length of N50 (bp)	2067		
Average length of all unigenes (bp)	1282		

### 2.3. Annotation of Non-Redundant Unigenes

Because the available genomic information for *Paulownia* is limited, a BlastX search with a threshold of 1.0 × 10^−5^ was performed to predict the functions of the all unigenes against six public databases (nr, nt, Swiss-Prot, KEGG (Kyoto Encyclopedia of Genes and Genomes), COG (Cluster of Orthologous Groups of proteins), and GO (Gene Ontology)). We found that 79,035 unigenes (68.66% of all unigenes) shared similarities with known sequences (Table 2).

**Figure 3:**
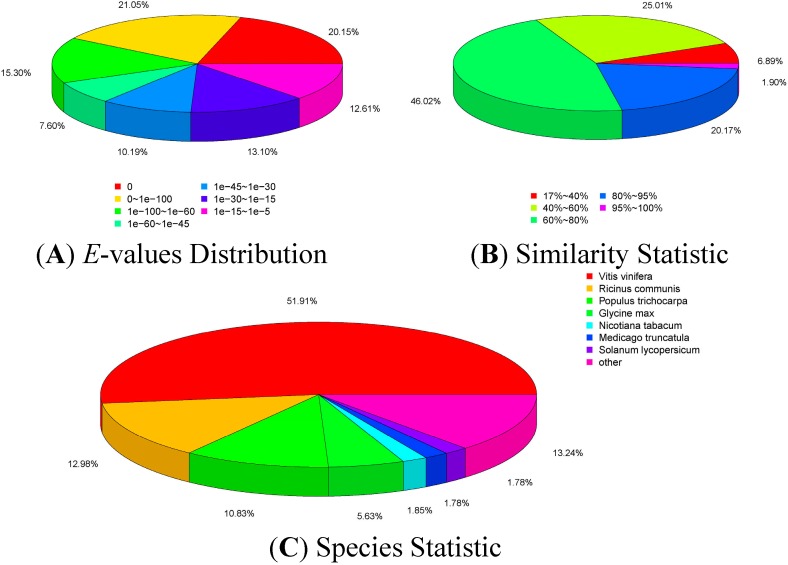
Distribution of *E*-values (**A**); similarity (**B**); and species (**C**) for the allunigene BLAST (Basic Local Alignment Search Tool) matches against the nr-protein database. For the BLASTX (Basic Local Alignment Search Tool, search protein database using a translated nucleotide query) matches the *E*-value cutoff was <1.0 × 10^−5^.

**Figure 4:**
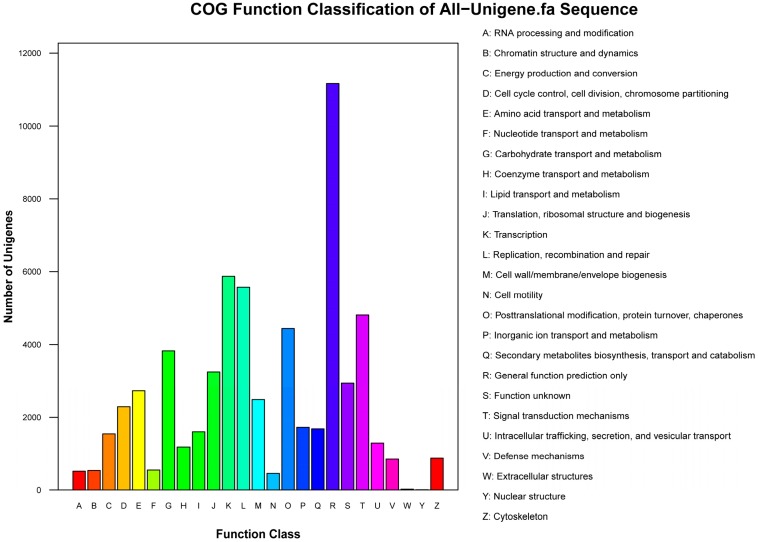
COG (Cluster of Orthologous Groups of proteins) classification of the *P. fortunei* all unigenes. 32,269 unigenes (28.0% of the total) were annotated and grouped into 25 specific categories.

**Figure 5:**
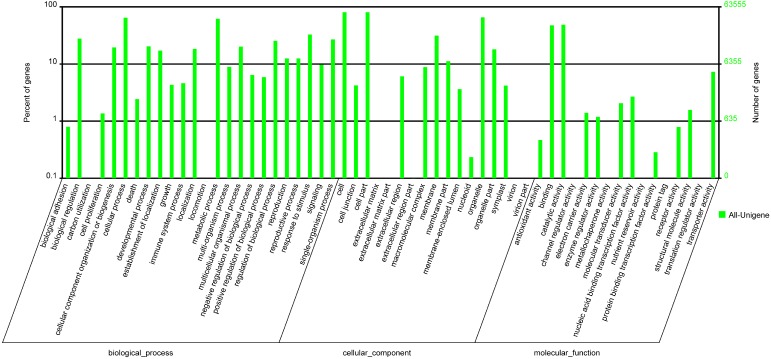
GO (Gene Ontology) classification of the *P. fortunei* all unigenes. 63,555 unigenes (55.2% of total) were annotated and categorized into 57 function groups.

Among the unigenes that matched known sequences, 74.3% showed strong homology (*E*-value < 1.0 × 10^−30^), and 25.7% showed some homology (1.0 × 10^−30^ < *E*-value < 1.0 × 10^−5^) with nr sequences (Figure 3A). The similarity distribution showed that 22.1% of the unigenes had similarities higher than 80%, and 77.9% had similarities of 17%–80% (Figure 3B). The species distribution showed that the unigenes had best matches to sequences from *Vitis vinifera* (51.9%), followed by *Ricinus communis* (13.0%), *Populus trichocarpa* (10.8%), and *Glycine max* (5.6%) (Figure 3C). Of the 115,116 all unigenes, 32,269 (28.0%) were assigned to 25 COG categories (Figure 4), 63,555 (55.2%) were assigned to at least one GO term (Figure 5), and 46,381 (40.3%) were mapped to 128 KEGG pathways (Table S1).

**Table 2:** Annotation of non-redundant unigenes of the transcriptome of *P. fortunei*.

Database	Number of Annotated Unigenes	Percentage of Annotated Unigenes (%)
nr	76,507	66.5
nt	69,981	60.8
Swiss-Prot	49,385	42.9
KEGG	46,381	40.3
COG	32,269	28.0
GO	63,555	55.2
All	79,035	68.7

### 2.4. Functional Classification of the Differentially Expressed Genes (DEGs)

**Figure 6:**
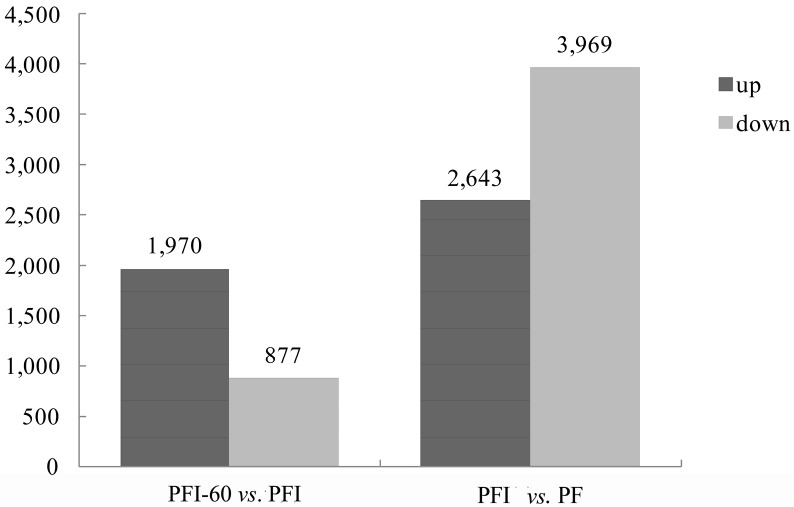
Statistics of the *P. fortunei* DEG (Differetially Expressed Genes) numbers in each comparison. PF represents the healthy wild-type sample of *P. fortunei*, PFI represents the sample of phytoplasma infected PF. PFI-60 represents the sample of 60 mg·L^−1^ MMS treated PFI.

In total, 7612 DEGs (Differentially Expressed Genes) were identified in the PFI *vs.* PF transcriptome comparison (2643 were up-regulated and 3969 were down-regulated), and 2847 DEGs were identified in the PFI-60 *vs.* PFI transcriptome comparison (1970 were up-regulated and 877 were down-regulated) (Figure 6). DEGs that were up-regulated in the PFI *vs.* PF comparison and down-regulated in the PFI-60 *vs.* PFI comparison were retrieved (397 DEGs), and DEGs that were down-regulated in the PFI *vs.* PF comparison and up-regulated in the PFI-60 *vs.* PFI comparison were also retrieved (912 DEGs). These 1309 DEGs were taken to be the PaWB disease-related DEGs analyzed in this study (Table S2). Top DEGs and DEGs related to *P. fortunei* PaWB disease were listed in Table 3. Several genes, including brassinosteroid insensitive 1-associated receptor kinase1 (*BAK1*), mitogen-activated protein kinase kinasekinase 1 (*MEKK1*), and WRKY transcription factor 29 (*WRKY29*) that may be involved in the plant-pathogen interaction pathway. Other plant-pathogen-related genes such as *PBS1*, which encodes a serine/threonine-protein kinase, and the transcription factor MYC2, were also identified among the DEGs. Adenylate isopentenyl transferase (*IPT*), two cytokinin trans-hydroxylases (*CYP735A*), and one cis-zeatin *O*-glucosyl transferase (*CISZOG*) that are involved in the biosynthesis of zeatin, abscisic acid receptor (*PYL*), ABA responsive element binding factor (*ABF*) and two lycopene cyclase (*CruA*) that involved in carotenoid biosynthesis pathways were altered during phytoplasma infection. DEGs involved in light signal transduction, including genes that encode GIGANTEA (*GI*), phytochrome-interacting factor 3 (*PIF3*), timing of CAB expression 1 (*TOC1*), circadian clock associated 1 (*CCA1*), and zinc finger protein CONSTANS (*CO*) were also identified.

**Table 3:** Top up- and down-regulated differetially expressed genes (DEGs) and DEGs related to *P. fortunei* PaWB disease. DEGs up-regulated in the PFI *vs.* PF comparison and meanwhile down-regulated in the PFI-60 *vs.* PFI comparison were retrieved. So were the DEGs down-regulated in the PFI *vs.* PF comparison and meanwhile up-regulated in the PFI-60 *vs.* PFI comparison. PF represents the healthy wild-type sample of *P. fortunei*, PFI represents the sample of phytoplasma infected PF. PFI-60 represents the sample of 60 mg·L^−1^ MMS treated PFI.

Gene ID	Gene Length (bp)	Up/Down *	Potential Gene Function
CL10743.Contig1	2028	Up	Inositol-3-phosphate synthase
CL10743.Contig2	1969	Up	Inositol-3-phosphate synthase
CL10796.Contig1	867	Up	AT5G14920
CL11352.Contig1	946	Up	MLP-like protein 34
CL3131.Contig1	2021	Up	Uncharacterized protein LOC100260696
CL6971.Contig3	1890	Up	Cytochrome P450
CL7523.Contig2	721	Up	Gibberellin induced protein
Unigene10760	611	Up	Plasma intrinsic protein 2.2
Unigene12341	1172	Up	Gibberellin induced protein
Unigene8595	323	Up	PIP2 protein
Unigene8675	1282	Up	UPF0301 protein Cpha266_0885-like isoform 1
CL13170.Contig1	858	Up	Transcription regulator
CL9733.Contig2	469	Up	Glycine-rich protein
CL3432.Contig3	2432	Up	Microtubule-associated protein TORTIFOLIA1
CL13388.Contig1	370	Up	Aquaporin PIP2
Unigene44849	866	Up	Cell wall-associated hydrolase
CL9691.Contig1	1215	Up	Alpha-expansin 2
Unigene43893	856	Up	Hypothetical protein MTR_8g040260
CL6181.Contig1	345	Up	Major latex-like protein
CL3545.Contig3	2147	Up	dof zinc finger protein DOF5.2
CL2544.Contig6	850	Down	Putative glycine-rich RNA binding protein
CL33.Contig2	2834	Down	WD repeat-containing protein C2A9.03
CL33.Contig3	5536	Down	WD repeat-containing protein C2A9.03
CL33.Contig4	5626	Down	WD-repeat protein
CL33.Contig5	2815	Down	WD repeat-containing protein C2A9.03
CL4119.Contig2	1113	Down	Cytochrome P450 72A1
CL4119.Contig5	2185	Down	CYP72A58
CL4739.Contig1	727	Down	Phylloplanin
CL4739.Contig2	610	Down	Phylloplanin
Unigene10248	4052	Down	WD repeat-containing protein C2A9.03
Unigene10249	1966	Down	WD repeat-containing protein C2A9.03
Unigene22577	953	Down	Unknown
Unigene24555	3910	Down	Unnamed protein product
Unigene32594	743	Down	Lipid transfer protein
Unigene28962	1800	Down	Glucose-6-phosphate/phosphate translocator 2
Unigene30587	4360	Down	GIGANTEA
Unigene30582	4161	Down	GIGANTEA
Unigene30581	4520	Down	GIGANTEA
CL4575.Contig2	2531	Down	1-Deoxy-d-xylulose-5-phosphate synthase
Unigene23241	3053	Down	WD repeat-containing protein C2A9.03
CL3202.Contig3	1081	Up	WRKY protein WRKY29
Unigene12008	1848	Up	Histidine kinase CRE1
CL8962.Contig1	915	Down	Histidine-containing phosphor transfer protein AHP
CL12775.Contig1	533	Up	Abscisic acid receptor PYL
CL2960.Contig1	818	Up	ABA responsive element binding factor ABF
Unigene27866	1130	Up	Adenylate isopentenyl transferase IPT
CL4119.Contig2	1848	Down	Cytokinin trans-hydroxylase CYP735A
CL4119.Contig5	2185	Down	Cytokinin trans-hydroxylase CYP735A
CL2490.Contig6	1644	Down	Serine/threonine-protein kinase PBS1
CL4203.Contig5	2362	Up	Circadian clock associated 1 CCA1
Unigene30581	4520	Down	GIGANTEA
Unigene10760	611	Up	Plasma intrinsic protein 2
Unigene14837	413	Up	Plasma membrane H^+^-ATPase LilHA2
Unigene44439	940	Up	Serine/threonine-protein kinase MEKK1
CL4362.Contig9	2426	Down	Transcription factor MYC2
Unigene19249	1046	Up	Zeatin *O*-glucosyl transferase CISZOG
CL4203.Contig5	2326	Up	Protein CCA1
CL4362.Contig9	2426	Down	Transcription factor PIF3
CL13348.Contig14	3472	Down	Brassinosteroid insensitive 1-associated receptor kinase1
CL4785.Contig7	2179	Down	Serine/threonine protein kinase PBS1
Unigene27866	1130	Up	Adenylate isopentenyl transferase IPT
Unigene19249	1046	Up	*Cis*-zeatin *O*-glucosyl transferase CISZOG
CL2374.Contig8	2053	Down	Lycopene cyclase CruA
Unigene28043	937	Up	Timing of CAB expression 1 TOC1
CL12024.Contig1	877	Up	Zinc finger protein CONSTANS

* Up or down regulation indicated the expression in the PFI *vs.* PF comparison. In the PFI-60 *vs.* PFI comparison, the tendencies were opposite.

Tounderst and the functions of the selected DEGs and also find disease related genes, we use GO terms and KEGG pathways to predict their functions. The DEGs were annotated with 48 terms (Table 4) under the three main GO categories, biological process, cellular component, and molecular function (Figure S2), and 535 of the DEGs were assigned to 83 KEGG pathways (Table 5 and Table S3).

**Table 4:** GO function analysis results for the *P. fortunei* DEGs.

Ontology	Class	Numbers of DEGs
Biological process	biological adhesion	32
	biological regulation	225
	cell proliferation	9
	cellular component organization or biogenesis	103
	cellular process	482
	death	44
	developmental process	173
	establishment of localization	119
	growth	31
	immune system process	43
	localization	128
	metabolic process	413
	multi-organism process	98
	multicellular organismal process	171
	negative regulation of biological process	45
	pigmentation	2
	positive regulation of biological process	42
	regulation of biological process	206
	reproduction	126
	reproductive process	126
	response to stimulus	301
	rhythmic process	17
	signaling	58
	sulfur utilization	2
Cellular component	cell	547
	cell junction	21
	cell part	547
	extracellular matrix	1
	extracellular region	78
	macromolecular complex	55
	membrane	230
	membrane part	63
	membrane-enclosed lumen	41
	organelle	392
	organelle part	102
	symplast	21
Molecular function	antioxidant activity	3
	binding	341
	catalytic activity	307
	electron carrier activity	22
	enzyme regulator activity	20
	molecular transducer activity	13
	nucleic acid binding transcription factor activity	25
	nutrient reservoir activity	1
	protein binding transcription factor activity	3
	receptor activity	3
	structural molecule activity	2
	transporter activity	53

**Table 5:** TOP 10 KEGG pathway analysis results for the *P. fortunei* DEGs.

#	Pathway	DEGs with Pathway Annotation	*p*-Value	*Q* Value	Pathway ID
1	Stilbenoid, diarylheptanoid and gingerol biosynthesis	30 (5.61%)	2.32 × 10^−18^	1.92 × 10^−16^	ko00945
2	Limonene and pinene degradation	22 (4.11%)	2.20 × 10^−13^	9.13 × 10^−12^	ko00903
3	Circadian rhythm-plant	30 (5.61%)	3.34 × 10^−12^	9.23 × 10^−11^	ko04712
4	RNA degradation	35 (6.54%)	3.30 × 10^−11^	6.85 × 10^−10^	ko03018
5	Carotenoid biosynthesis	21 (3.93%)	8.94 × 10^−10^	1.48 × 10^−8^	ko00906
6	Biosynthesis of secondary metabolites	92 (17.2%)	3.82 × 10^−7^	5.29 × 10^−6^	ko01110
7	Tryptophan metabolism	11 (2.06%)	2.70 × 10^−6^	3.21 × 10^−5^	ko00380
8	Porphyrin and chlorophyll metabolism	15 (2.8%)	6.78 × 10^−6^	6.85 × 10^−5^	ko00860
9	Monoterpenoid biosynthesis	7 (1.31%)	7.43 × 10^−6^	6.85 × 10^−5^	ko00902
10	Isoflavonoid biosynthesis	6 (1.12%)	3.32 × 10^−5^	2.76 × 10^−4^	ko00943

### 2.5. Confirmation of the RNA-Seq Expression Data by qRT-PCR

**Figure 7:**
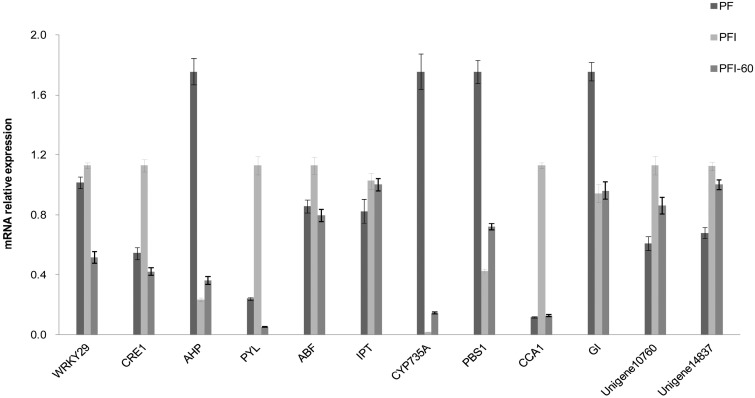
qRT-PCR analysis of candidate *P. fortunei* PaWB resistant genes. (The following genes were validated: WRKY transcription factor 29 (*WRKY29*), WRKY protein; *CRE1*, histidine kinase; *AHP*, histidine-containing phosphor transfer protein; *PYL*, abscisic acid receptor; *ABF*, ABA responsive element binding factor; *IPT*, adenylate isopentenyl transferase; *CYP735A*, cytokinin trans-hydroxylase; *PBS1*, serine/threonine-protein kinase; *CCA1*, circadian clock associated 1; *GI*, GIGANTEA; *Unigene10760*, plasma intrinsic protein 2; *Unigene14837*, plasma membrane H^+^-ATPase LilHA2. 18S rRNA was used as the internal reference gene. Standard error of the mean for three technical replicates is represented by the error bars. PF represents the healthy wild-type sample of *P. fortunei.* PFI represents the sample of phytoplasma infected PF. PTFI-60 represents the sample of 60 mg·L^−1^ MMS treated PFI).

To confirm the results of the transcriptome sequencing analysis, qRT-PCR was performed on 12 selected candidate DEGs. The primers used for the qRT-PCR analysis, potential gene functions, and amplicon sizes are shown in Table S4. cDNA fragments from the samples used for the transcriptome sequencing were used as templates. The expression patterns revealed by qRT-PCR (relative expressed level) of the allunigenes were consistent with their expression predicted by analysis of the DEG sequences (Figure 7). This result validated the gene expression variations obtained from our analysis of the unigene sequences.

## 3. Discussion

The molecular mechanisms involved in the interaction between the PaWB phytoplasma and *P. fortunei* are largely unknown. Using RNA-Seq technology, we identified *Paulownia* genes and pathways whose expression profiles were altered among healthy, infected, and MMS-treated *P. fortunei* samples. Some of the differentially expressed sequences showed no similarity with sequences in the public databases, indicating that they may have novel important functions in the phytoplasma infection process and that they may be specific to *P. fortunei*.

Previously, we proposed a hypothesis about the plant-phytoplasma compatible interactions of *P. tomentosa* × *P. fortunei* [13]. Here, we retrieved the DEGs related to PaWB disease in *P. fortunei* and found that similar mechanisms may be involved. Based on the annotations of the final PaWB disease-related DEGs, we classified the response of *P. fortunei* to phytoplasma infection at the cellular and molecular levels. At the cellular level, the responses included modulation of the membrane system, modification of the cell wall, and alternation of the cell cycle and division. At the molecular level, the responses included transient alterations of the membrane ionic permeability such as H^+^, Ca^2+^, and K^+^. This response might be expected to initiate the synthesis and release of secondary messengers, including reactive oxygen species (ROS) (e.g., H_2_O_2_) that play significant roles in plant-pathogen reaction systems [15]. Elevated concentrations of ROS are noxious to plants, especially in some areas of the cells. Thus, antioxidants, such as cytochrome P450, are a necessary part of the defense response. High levels of the second messengers can cause a protein phosphorylation cascade reaction as has been reported in a similar study by [16]. Further, transcription factor (TF) expression might be activated, which would then initiated the expression of the related stress-induced genes, such as the enzymes involved in detoxin processes, the transporters of ions, the regulators of other TFs, protein kinases, and phosphatases. These data, to some extent, supported our previous study [13] and provided insights into the molecular mechanism of PaWB disease in *P. fortunei*.

In addition to the mechanisms described above, three different groups of DEGs associated with the response of *P. fortunei* to PaWB disease were identified.

### 3.1. DEGs Related to Plant-Pathogen Interaction

**Figure 8:**
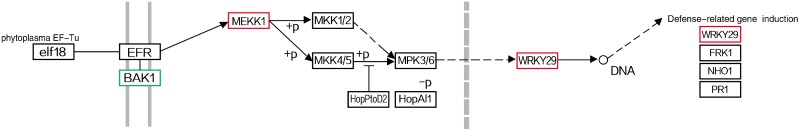
Elongation factor Tu (EF-Tu) response pathway in *P. fortunei*. Genes in boxes are differently expressed. Red boxes indicate up-regulated or induced genes in the infected seedlings. Green boxes indicate down-regulated ones.

It has been reported that the inducible plant defense response to pathogens is multilayered and at least two stages are involved [17,18]. In the first stage, plant pattern recognition receptors trigger the recognition of pathogen-associated molecular patterns (PAMPs) [19], resulting in PAMP-triggered immunity. The second stage is initiated by the recognition of pathogen virulence proteins (effectors) or their activities by plant disease resistance (R) genes and the consequence is effector-triggered immunity [20]. PAMPs include the intracellular translation elongation factor Tu (EF-Tu). A conserved domain in EF-Tu, which can trigger PAMP-triggered immunity, has been recognized in *Brassicaceae* [21,22]. Phytoplasmas harbor genes that encode EF-Tu, and the products may induce PAMP-triggered immunity. In our study, we identified several genes, including *BAK1*, *MEKK1*, and *WRKY29* that may be involved in the plant-pathogen interaction pathway. The pathway is shown in Figure 8. WRKY29 can induce the expression of defense-related genes, which include WRKY29 itself. Other plant-pathogen-related genes were also identified among the DEGs, such as *PBS1*, which encodes a serine/threonine-protein kinase, and the transcription factor MYC2.

### 3.2. DEGs Related to Hormone Biosynthesis

During phytoplasma infection, plant hormones have been reported to be involved in symptom formation, such as witches’ broom, smaller leaves, and short internodes [23,24]. Previous studies have found that elevated cytokinin (CK) levels can induce such symptoms. CKs can be synthesized through two major pathways, cis-zeatin and trans-zeatin, both of which need the presence of zeatin. Our analysis revealed several genes, including one *IPT*, two *CYP735A*, and one *CISZOG* that are involved in the biosynthesis of zeatin, which were differentially expressed in the process of phytoplasma infection (Table S1). It has been reported that in trans-zeatin biosynthesis, IPT is the first and key enzyme in the pathway [25,26,27]. Up-regulation of *IPT* in *P. fortunei* would elevate the concentration of CKs while down-regulation of *CYP735A* would probably affect the conversion of isopentenyladenine (iP)-nucleotide to tZ nucleotide. Thus, most CKs in *P. fortunei* may exist in the iP-type form. Furthermore, *CRE1* and *AHP* that participate in the metabolism of CK were found to be differentially expressed. The overall effect of the genes described above would be to trigger cell division and shoot initiation.

Auxins have been reported to have anti-phytoplasma activity [28,29]. In our study, the genes related to auxin were found to be slightly down-regulated. As a result, the ratio of CK to auxin would be elevated, which could cause apical dominance and the formation of lateral buds (a symptom of witches’ broom), as reported previously [30,31].

The stress-related hormone abscisic acid (ABA) has been shown to inhibit shoot growth and plant stem elongation [32,33]. We found among the DEGs associated with the carotenoid biosynthesis pathways that the expressions of *PYL*, *ABF* and two *CruA* were altered. These genes encode carotenoid precursors for ABA biosynthesis [34], which may induce stomatal closure and seed dormancy. Therefore, the formation of shorter internodes and smaller leaves in PaWB-infected plants may be associated with an increase in ABA.

In summary, we identified several genes related to CK and ABA biosynthesis that were differentially expressed after phytoplasma infection. Typical syndromes of phytoplasma infection seem to be associated with these genes. Therefore, CK and ABA may be important functional hormones in the response of *Paulownia* to witches’ broom.

### 3.3. DEGs Related to Circadian Rhythms

**Figure 9:**
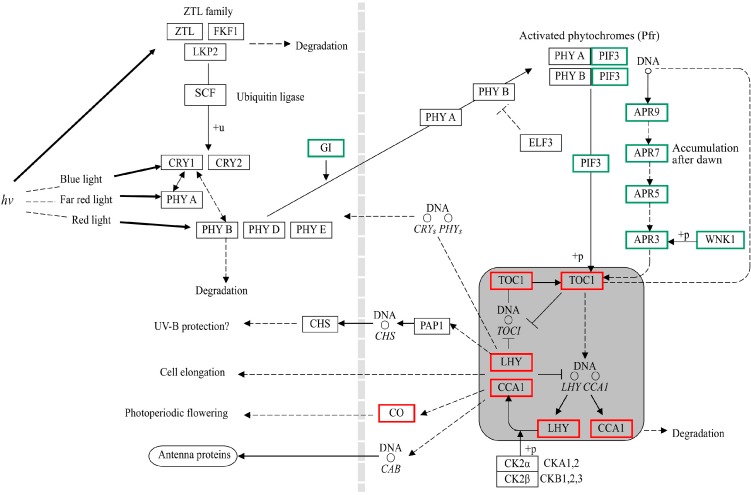
Circadian rhythm pathway in *P. fortunei*. Genes in boxes are differently expressed. Red boxes indicate up-regulated or induced genes in the infected seedlings. Green boxes indicate down-regulated ones.

Light provides a variety of signals for growth and development in higher plants. In the present study, several DEGs involved in light signal transduction were identified. The expressions of these genes were altered in the PFI samples compared with the PF samples. After MMS treatment, the alternations in expression returned to the expressions observed in the healthy (PF) samples (Figure 9).

Plants have evolved subtle photoreceptors system, such as phytochromes, cryptochromes, and phototropins, that regulate various light responses (e.g., blue light, far red light, or red light) through their interaction with different signaling proteins, among which the basic helix–loop–helix protein PIF3 is the most well-characterized [35]. This light-dependent regulator can induce light-regulated genes by binding to the phytochromes PHYA and PHYB. It has been proposed that PIF3 may act not only as a positive TF for chloroplast development, but also as a negative regulator for hypocotyl elongation [36]. A study on *Arabidopsis thaliana* seedlings reported that under diurnal light/dark conditions, PIF3 could target growth-related genes, and thus could also be considered a growth promoter [37]. In the present study, a gene that coded PIF3 was found to be down-regulated in PFI *vs.* PF and up-regulated in PFI-60 *vs.* PFI, which suggested that decreased light reception in the phytoplasma-infected *Paulownia* may contribute to the abnormal development of certain organs and decrease their sizes.

Changes in the gene expression patterns may alter the circadian rhythm and allow the plant to sense environmental light changes during the day/night cycle. Previous studies have showed that genes encoding *TOC1*, *MYB*, *CCA1*, and *LHY* work together as an autoregulatory loop, which could connect morning- and evening-phase circuits [38]. For example, it has been reported that when the expressions of *LHY* and *CCA1* rose in the morning the expression of the evening-expressed *TOC1* fell and, conversely, when the expressions of *LHY* and *CCA1* fall, the expression of *TOC1* rises [39]. In the present study, two genes that encode *TOC1* and *LHY*/*CCA1*, respectively, were altered in both the PFI *vs.* PF and PFI-60 *vs.* PFI comparisons and were also predicted to be involved in the circadian rhythm pathway in the KEGG analysis. Several metabolic developmental processes in plants have been reported to show a circadian rhythm in leaf movement and in stomata opening, and this has been correlated with the CO_2_ assimilation rate [40]. The DEGs related to circadian rhythm in the PFI sample may decrease the photosynthesis rate in the PFI and PFI-60 samples; thus, changing the growth rate of the plants.

## 4. Experimental Section

### 4.1. Preparation of Plant Material

All of the biological material used in this study was obtained from the Institute of *Paulownia*, Henan Agricultural University, Zhengzhou, Henan Province, China. Tissue culture seedlings of *P. fortunei*, PF (healthy; three individuals) and PFI (PaWB-infected; six individuals) were cultured for 30 days before being clipped from the roots. The PFIs were transferred into 100-mL triangular flasks containing 1/2 MS culture medium [8] containing 25 mg·L^−1^ sucrose and 8 mg·L^−1^ agar (Sangon, Shanghai, China) with 0 or 60 mg·L^−1^ MMS. The PFs were transferred into 100-mL triangular flasks containing 1/2 MS medium without MMS as the control. At least three parallel samples were prepared for each condition. All the samples were first cultured at 20 °C in the dark for 5 days. After that, they were cultured under 25 ± 2 °C and 130 µmol·m^−2^·s^−1^ intensity light for 16 h every day.

### 4.2. Construction of cDNA Libraries of P. fortunei

For each condition, approximately 8 mg of leaves were homogenized in liquid nitrogen with a pestle. Total RNA was extracted from the cells using TRIzol reagent (Invitrogen, Carlsbad, CA, USA), followed by RNA purification using the RNeasyMiniElute Cleanup Kit (Qiagen, Dusseldorf, Germany) according to the manufacturer’s protocol. With a NanoVue UV–Vis spectrophotometer (GE Healthcare Bio-Science, Uppsala, Sweden), RNA was quantified by measuring absorbance at 260 nm. Absorbance ratios of OD_260/280_ and OD_260/230_ were used to assess the purity of all the RNA samples. The integrity of the RNA was checked by 1% agarose gel electrophoresis. After total RNA extraction and DNase I treatment, magnetic beads with Oligo (dT) were used to isolate the mRNA. The mRNA was mixed with fragmentation buffer to obtain short fragments. cDNA was synthesized using the mRNA fragments as templates. The short cDNA fragments were purified and resolved with EB buffer for end reparation and single nucleotide A (adenine) addition. Then, the short fragments were connected with adapters. Suitable fragments were used as the templates for PCR amplification. An Agilent 2100 Bioanalyzer (Agilent Technologies, Palo Alto, CA, USA) and ABI StepOnePlus Real-Time PCR System (ABI, New York, NY, USA) were used to quantify and assess the quality of the sample library. Finally, the library was sequenced using IlluminaHiSeq™ 2000 (Illumina, San Diego, CA, USA).

### 4.3. Bioinformatics Analysis

High-throughput RNA-seq was performed by Illumina RNA sequencing in Beijing Genomics Institute-Shenzhen (BGI-Shenzhen, Shenzhen, China). Base calling was used to transform the image data output from the sequencing machine into sequence data. Two software programs were used in this process: a real-time analyzer and the off-line basecaller (OLB). The real-time analyzer software, which was installed on the HiSeq™ 2000 sequencer, can analyze the results while sequencing, and convert the image files into bcl files after analysis. The bcl files were output to the mainframe computer that ran the OLB software (version 1.9.4) to convert the bcl files into sequence data files. The input and output parameters (setupBclToQseq.py-b path/to/BaseCalls-o path/to/output/directory) used were the ordinary ones and no special parameters were involved. After that, another parameter (--ignore-missing-stats) was added to ignore the situations of missed files. The sequence data that were obtained were the raw reads and these data were stored in fastq format. The raw reads included low quality reads that contain adapters, and unknown or low quality bases. These contaminants were filtered out using the filter_fq program to produce the clean reads. The clean reads have been deposited in the NCBI Short Read Archive (SRA) database under accession number SRP040429. The Trinity short reads assembly software [41] was used to carry out *de novo* assembly of the clean reads. The assembled sequences were called unigenes. When multiple samples from the same species are sequenced, the unigenes from each assembly can be processed with sequence clustering software to remove sequence splicing and redundancy to acquire non-redundant unigenes that are as long as possible. Clustering of our unigene sequences generated clusters that contained several unigenes that shared more than 70% sequence similarity, these clusters were named using the prefix CL, and the remaining unigenes that did not form clusters were the singletons, which were named using the prefix Unigene.

Blastx alignments (*E*-value < 1.0 × 10^−5^) were performed between the assembled unigenes and sequences in the following protein databases: NCBI’s non-redundant protein database (NR), Swiss-Prot, Kyoto Encyclopedia of Genes and Genomes (KEGG), and clusters of orthologous groups (COG). The sequence directions were decided based on the best alignment results over all these databases. When different databases generated conflicting results, the sequence direction was assigned based on a priority order of NR, Swiss-Prot, KEGG, and COG. When a unigene sequence could not be aligned to any of the sequences in the four databases, ESTS can [42] was used to decide its direction.

### 4.4. Database Searches

The CL and Unigene sequences were first aligned to the protein databases NR, Swiss-Prot, KEGG, and COG (*E*-value cutoff was < 1.0 × 10^−5^) using blastx, and to NCBI’s nucleotide NT database (*E*-value cutoff < 1.0 × 10^−5^) using blastn. The protein sequences that shared the highest sequence similarity with the aligned unigenes were retrieved along with their Gene Ontology (GO) functional annotations. The KEGG database provided pathway annotations for the matched unigenes. The unigenes were also aligned to COG to help predict and classify the possible unigene functions.

### 4.5. Unigene Gene Ontology (GO) Classification

We used the Blast2GO program [43] to retrieve the GO annotations from the NR database for each of the matched unigenes. To understand the distribution of the functions of the genes in *P. fortunei* at the macro level, we used the WEGO software [44] to perform a GO functional classification for each unigene annotation.

### 4.6. Protein Coding Sequence (CDS) Prediction

The unigenes that matched sequences in the NR and Swiss-Prot databases were also aligned to KEGG and COG. Protein sequences with the highest ranks in the blast alignments were retrieved and used to decide the CDS of each unigene. The predicted CDSs were translated into amino sequences using the standard codon table to obtain both the nucleotide sequence (5'–3') and amino sequence for each unigene CDS. ESTS can [42] was used to predict the nucleotide sequence (5'–3') direction and the amino sequence for the unigenes that could not be aligned to any of the four databases.

### 4.7. Unigene Expression Analysis

We used the FPKM (fragments per kilobase of transcript per million mapped reads) method [45] to normalize read counts and determine the values of the expressed genes of the *Paulownia* transcriptomes, PFI *vs.* PF and PFI-60 *vs.* PFI. We have developed a rigorous algorithm based on Audic *et al.* [46] to identify DEGs between two samples. The null hypothesis and alternative hypothesis to identify DEGs were defined as: *H*_0_, a gene with the same expression level in two samples; and *H*_1_, a gene with different expression levels in two samples.

If *x* is the number of fragments that can map uniquely to gene A, then for each transcript representing a small fraction of the library, *p*(*x*) will closely follow the Poisson distribution [46] as:

(1) $$p\left(x\right)=\frac{e^{-\lambda }{\text{λ}}^{x}}{x!}$$

where λ is the real transcripts of the gene.

If the total number of fragments in sample 1 is *N1*, and total number of fragments in sample 2 is *N2*, and if gene A has *x* fragments in sample 1 and *y* fragments in sample 2, then the probability of gene A being expressed equally in the two samples can be calculated using the following formula [45]:

(2) $$2\sum_{i=0}^{\text{y}}\text{p}(i|\text{x})(\text{while}\sum_{i=0}^{\text{y}}\text{p}(i|\text{x})\leq 0.5)$$

or

(3) $$2(1-\sum_{i=0}^{\text{y}}\text{p}(i|\text{x}))(\text{while}\sum_{i=0}^{\text{y}}\text{p}(i|\text{x})\geq 0.5)$$

where

(4) $$p\left(i|x\right)={\left(\frac{{\text{N}}_{2}}{{\text{N}}_{1}}\right)}^{i}\frac{(x+i)!}{x!\;i!(1+{\left(\frac{{\text{N}}_{2}}{{\text{N}}_{1}}\right)}^{\left(\;\text{x}+i+1\right)}}$$

After performing thousands of hypothesis tests, we found that a significant *p*-value for an individual test was not enough to guarantee a low false discovery rate (FDR). Therefore, we used a multiple testing correction method for each individual hypothesis testing to guarantee the FDR was low overall.

FDR control is a statistical method used in multiple hypothesis testing to correct for *p*-value. In practical terms, the FDR is the expected FDR; for example, if 1000 observations were predicted experimentally to be different and the maximum FDR for these observations was 0.1, then 100 of these observations would be expected to be a false discovery, See [47] for details of this method. We then used the FDR along with the ratio of FPKMs for the two samples. The smaller the FDR is and the larger the ratio is, the larger the difference is in the expression levels between the two samples. A threshold FDR ≤1.0 × 10^−3^ and an absolute value of log2 Ratio >2 were used to judge the significance of differences in gene expression.

### 4.8. GO Functional Enrichment Analysis of the DEGs

All the DEGs were first mapped to terms in the GO database and the numbers of genes annotated with each GO term were calculated to obtain a gene list and the numbers of genes for every assigned GO term. To identify significantly enriched GO terms among the DEGs, a hypergeometric test was used. The formula used to calculate the *p*-value in this hypothesis test was:

(5) $$P=1-\sum_{i=0}^{m-1}\frac{\left(\begin{array}{c} M\\ i \end{array}\right)\left(\begin{array}{c} N-M\\ n-i \end{array}\right)}{\left(\begin{array}{c} N\\ n \end{array}\right)}$$

where *N* is the number of all the genes with GO annotations, *n* is the number of DEGs in *N*, *M* is the number of all genes that are annotated to a certain GO term, and *m* is the number of DEGs in *M*. The *p*-value calculated after a Bonferroni correction of ≤0.05 was used as the threshold. GO terms that fulfilled this condition were defined as significantly enriched in the DEGs against the whole transcriptome background. The GO functional enrichment analysis was used to recognize the main biological functions of the DEGs and to integrate the clustering analysis of the expression patterns to easily obtain the expression patterns of the DEGs annotated with GO terms.

### 4.9. Kyoto Encyclopedia of Genes and Genomes (KEGG) Pathway Analysis of the DEGs

Pathway enrichment analysis was used to retrieve significantly enriched pathways in the DEGs against the whole transcriptome background. A similar formula to the one used to calculate the *p*-value in the GO analysis was used. A *q*-value was defined as the FDR analog of the *p*-value. After multiple testing and correction, we choose a *q*-value of ≤0.05 to identify the pathways that were significantly enriched in the DEGs. The parameters of all the software used in this study were shown in Table S5.

### 4.10. Quantitative Real-time PCR (qRT-PCR) Analysis

The RNA samples from the leaves of the PF, PFI, and PFI-60 samples were extracted with Trizol (Sangon, Shanghai, China). The RNA was then precipitated with isopropanol. Purified and concentrated RNA was denatured and first-strand cDNAs for all the samples were synthesized using a PrimeScript RT reagent Kit (Takara, Dalian, China). Potential genes related to PaWB disease resistance were chosen. Primers were designed with Beacon Designer, version 7.7 (Premier Biosoft International, Ltd., Palo Alto, CA, USA). The cDNAs were then amplified in a Bio-Rad CFX96TM Real-Time System (Bio-Rad, Hercules, CA, USA) with SYBR Premix Ex Taq TM II (Takara, Dalian, China). The following PCR parameters were used: 50 °C for 2 min, 95 °C for 30 s, followed by 40 cycles of 94 °C for 15 s and 60 °C for 1 min. Three replicates were analyzed for each gene. The average threshold cycle (*C*_t_) was normalized and the relative expression changes were calculated using the 2^−∆∆*C*t^ method. The 18S rRNA of *Paulownia* was chosen as an internal reference gene for normalization.

## 5. Conclusions

In summary, this investigation provides the first transcriptomic analysis of phytoplasma-infected *P. fortunei* and the transcriptome data have enriched our knowledge of *P. fortunei*. Further, the analysis of the DEGs in healthy, phytoplasma-infected, and MMs-treated phytoplasma-infected *Paulownia* seedlings revealed a number of candidate genes and inherent pathways related to the PaWB disease symptoms, which has shed some light on the molecular mechanisms associated with this biological process. Although a large number of novel disease-related DEGs remain unannotated, the current study has established a substantial platform for in-deep studies into the mechanisms in *Paulownia* that are affected by the biotic stress caused by phytoplasma infection. There are common mechanisms in the process of photoplasma infection between different *Paulownia* species. However, some different mechanisms and a lot of DEGs from other *Paulownia* species have also been found. Further, this study provides new insights into the response and resistance of *P. fortunei* against PaWB disease.

## Supplementary Materials

Supplementary materials can be accessed at: http://www.mdpi.com/1422-0067/15/12/23141/s1.
